# Into the Unknown: A Case of New-Onset Cardiomyopathy in a Patient Treated With Recently Approved Tyrosine Kinase Inhibitor, Pralsetinib

**DOI:** 10.7759/cureus.15441

**Published:** 2021-06-04

**Authors:** Alexa Papaila, Alexis T Jacobson

**Affiliations:** 1 Internal Medicine, Brown University, Providence, USA

**Keywords:** tyrosine kinase receptor inhibitors, pralsetinib, molecularly targeted therapy, drug-induced cardiomyopathy, systolic heart failure

## Abstract

With the rapid development and adoption of novel anti-cancer therapeutics, physicians commonly encounter cancer patients on regimens with recently approved drugs for which information about rare or long-term side effects may not be available. In this case, we present a young woman with cholangiocarcinoma who was treated with the rearranged during transfection (RET)-selective tyrosine kinase inhibitor (TKI), pralsetinib, and presented to the hospital with shortness of breath. We review her diagnosis of new-onset systolic dysfunction as a possible sequela of her TKI therapy to encourage ongoing efforts to enhance provider familiarity with the side effects of this important and increasingly prescribed drug class.

## Introduction

With the rapid emergence of novel molecularly targeted pharmacotherapeutics, nononcologic practitioners are faced with the task of maintaining at least a peripheral familiarity with these drug classes and their adverse effects. For those interested in cardiology, this is particularly important in that several biologics and chemotherapy agents [e.g., alkylating agents, anthracyclines, interferon alfa, monoclonal antibodies, tyrosine kinase inhibitors (TKIs)] exhibit direct cardiotoxic effects [1-4]. TKIs are increasingly prescribed and have drastically improved the quality of life and survival in patients with solid tumors [5]. However, TKIs are commonly linked to cardiomyocyte apoptosis and fibrosis, systolic dysfunction, QT prolongation, and pericardial effusion [1-4]. Pralsetinib is one such TKI that was recently approved by the U.S. Food and Drug Administration (FDA) for use in patients with non-small cell lung cancer and thyroid cancers. Pralsetinib is a commonly used off-label for patients with gastrointestinal cancers and the rearranged during transfection (RET) gene mutation. Here we report a case of systolic dysfunction in a patient with RET fusion + cholangiocarcinoma treated with pralsetinib.

## Case presentation

A 36-year-old woman with stage IV cholangiocarcinoma with prior common bile duct stent placement presented from her oncology clinic with dyspnea, fever, and malaise. The patient had no other medical conditions or cardiovascular risk factors. She was initially diagnosed with cholangiocarcinoma five months earlier and initiated on combination chemotherapy with folinic acid, fluorouracil, and oxaliplatin (FOLFOX). She experienced disease progression after four cycles of FOLFOX and the regimen was stopped. She was then found to have the RET fusion gene and was started on the RET-selective TKI, pralsetinib.

**Figure 1:**
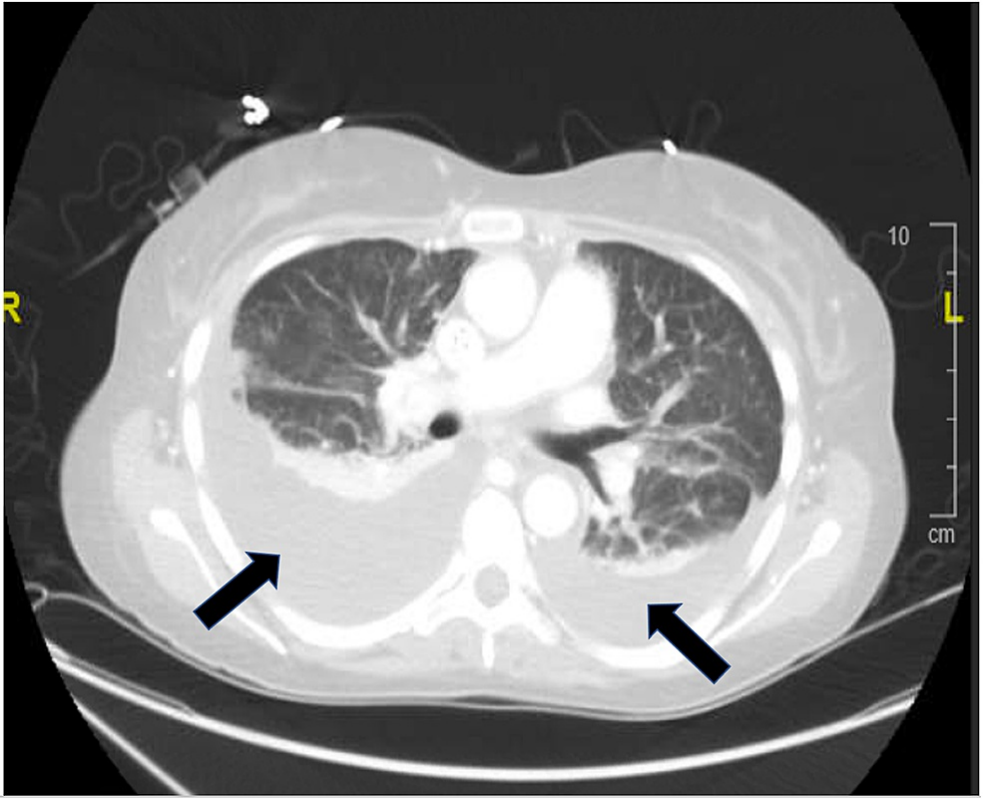
CT chest, transverse section, demonstrating bilateral pleural effusions (black arrows, right larger than left).

**Figure 2:**
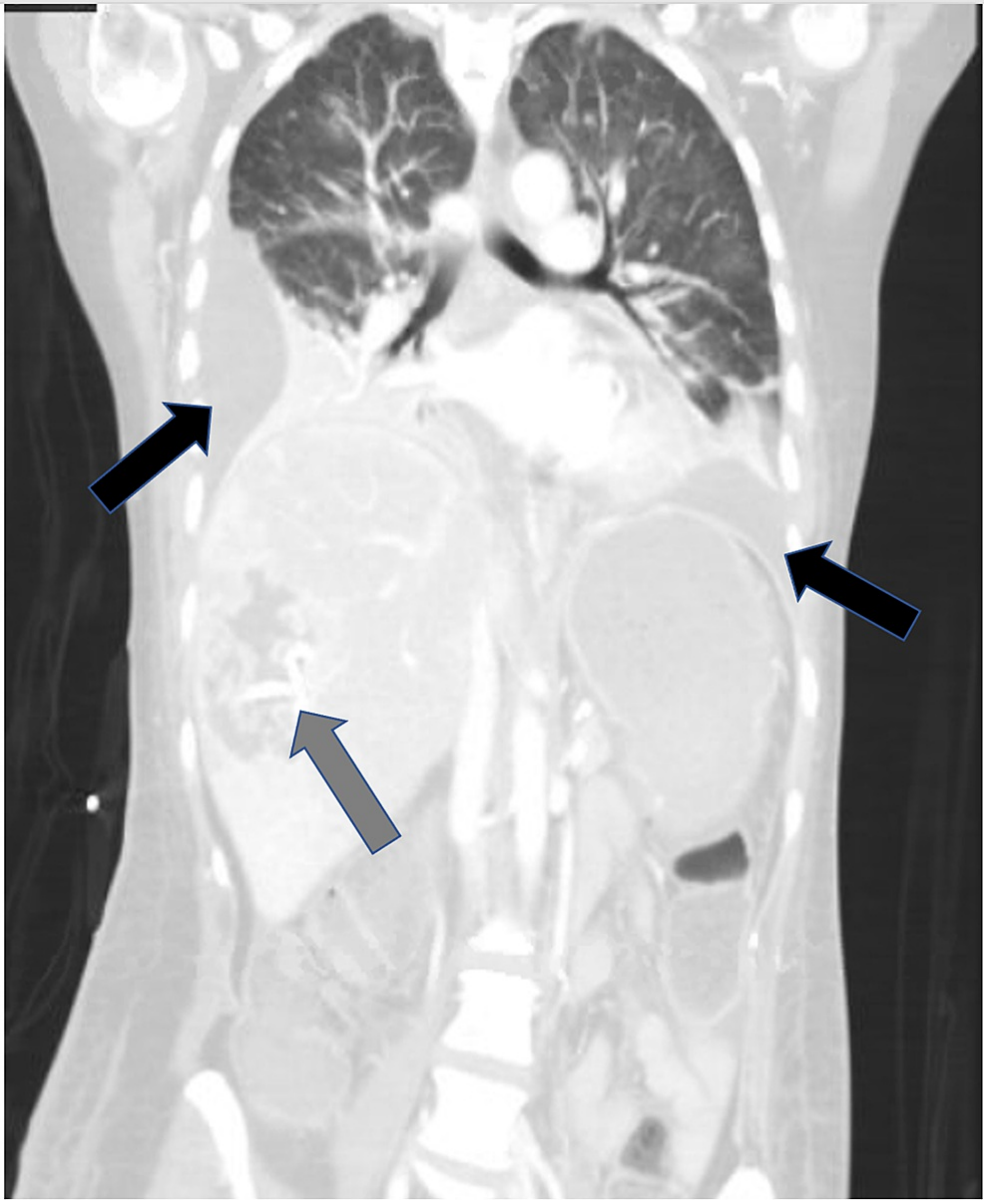
CT chest and abdomen, coronal section, demonstrating bilateral pleural effusions (black arrows), right hemidiaphragm elevation secondary to hepatomegaly with drain (gray arrow) in right hepatic lobe abscess.

On arrival, she was tachypneic to the 40s and hypoxemic with oxygen saturations in the low 80s. She was tachycardic with heart rates reaching 150 beats per minute, normotensive, and febrile to 103.4°F. She was ill-appearing, lethargic, pale with warm, dry extremities and moderate epigastric tenderness. Lactate was 5.1 mEq/L, serum creatinine was newly elevated to 1.78 mg/dL, and she had a moderate elevation of her baseline transaminitis and hyperbilirubinemia. She had mild leukocytosis with 14,600 per mm3 white blood cells and bandemia to 13%. Her hemoglobin was 6.6 g/dL from her baseline of 9.0 g/dL. Electrocardiogram (ECG) revealed sinus tachycardia. CT of the chest, abdomen, and pelvis revealed interlobular septal thickening of the lungs with bilateral pleural effusions and a 6.3 cm x 6.7 cm x 8.3 cm multiloculated fluid collection in the right hepatic lobe (Figures 1-2). 

After percutaneous hepatic abscess drainage and drain placement, repeat chest CT revealed worsening pulmonary effusion necessitating therapeutic thoracentesis with chest tube placement with subsequent near-resolution of her effusion. However, the patient continued to experience significant respiratory distress and hypoxemia. Repeat ECG was unchanged, but a transthoracic echocardiogram (TTE) was performed which noted new global systolic dysfunction (left ventricular ejection fraction 38% from her baseline of 60%). Her respiratory distress was ultimately attributed to bilateral parapneumonic effusions complicated by new-onset heart failure. After discussion with the consulting cardiology and oncology teams, her pralsetinib was held. She was diuresed aggressively with improvement in her respiratory function and initiated on guideline-directed medical therapy for heart failure prior to discharge. Multigated acquisition scan revealed partial recovery of her left ventricular ejection fraction to 46% one month after drug cessation. 

## Discussion

Pralsetinib is a novel TKI designed for selective RET-kinase inhibition. While pralsetinib is known to cause transaminitis and cytopenias, the FDA has not identified systolic dysfunction as a common side effect [6]. TKIs are associated with cardiotoxicity, especially among later generation TKIs that nonselectively inhibit the potent angiogenic factor vascular endothelial growth factor (VEGF) and interrupt the functional integrity of the heart [2, 4, 7]. Pralsetinib's RET-kinase selectivity theoretically reduces the risk of cardiotoxicity most often associated with nonselective TKIs and VEGF inhibition [7]. However, pralsetinib has exhibited in vitro activity against VEGFR-2, Janus kinase (JAK) inhibitors, and other compounds. The relative but incomplete selectivity for RET kinase may reduce but not eliminate the risk of cardiotoxic effects [7]. 

To date, there are no available reports on the development of heart failure in patients taking pralsetinib [6]. In the ongoing phase 2 trial studying adverse effects of pralsetinib, the majority of adverse events recorded according to the National Cancer Institute Common Terminology Criteria for Adverse Events were grade 1 or grade 2 events. In our patient with heart failure with a reduction of greater than 20% from her baseline, this would qualify as a grade 3 event. As observed in similar cases where cessation of targeted therapies improved patient's symptoms [8], our patient's improvement in respiratory function and partial recovery of systolic function after drug cessation reemphasize the need for ongoing risk-benefit conversations about drug continuation. It is possible that our patient’s cardiac dysfunction may not be solely attributable to the initiation of pralsetinib. For example, drugs like fluorouracil (which she received prior to her treatment with pralsetinib) are associated with cardiotoxicity. However, our patient's symptoms did not develop until several weeks after discontinuation of fluorouracil and subsequently several weeks after initiation of pralsetinib. It is important to note that we do not yet have long-term data on the cardiotoxic effects of these novel drugs and should be prudent contributors to surveillance data to ensure providers across all fields have reliable data to reflect on potential etiologies contributing to patient’s presentations.

## Conclusions

This case of new-onset heart failure in a patient treated with pralsetinib demonstrates an important potential adverse effect that has not yet been described. In addition to expanding informed consent to include the possibility of heart failure in patients considering treatment with pralsetinib, this case demonstrates the importance of multi-disciplinary involvement (oncologists, pulmonologists, and cardiologists) in the care of critically ill cancer patients on novel cancer drugs. 
